# The Jamaica Physical Journal

**Published:** 1835-07-01

**Authors:** 


*The Jamaica Physical Journal.* Edited by James Paul, Esq. Kingston, Saturday, September 6, 1834. 8vo. pp. 120.

This Number commences with a review of Hahnemann's Organon, extracted from the first Number of our Journal; this is followed by other quotations from English periodicals, &c., and at p. 69 we arrive at the original papers.

Dr. Miller, in an Essay read before the College of Physicians and Surgeons of Jamaica, has some remarks on the use of ipecacuanha in restraining hemorrhage. He has given it with great success in uterine hemorrhage, in the dose of two grains every hour, made into a pill with laudanum; three pills are usually sufficient. In other hemorrhages he prefers the acetate of lead.

He next mentions the Malacia, or Pica Africanorum. This is the disease of the Negroes which is supposed to arise from eating dirt, and closely resembles the malady described by Andral under the name of Anemie génerale. (*Anat. Path. tom.* i. p. 80.) The palpitations of the heart and dyspnœa which attend this disease, and are called by the Negroes pain of stomach, are highly distressing, and sometimes threaten speedy dissolution.

"Recollecting some cases related by Dr. Darwin in his Zoonomia, in which he had adopted a practice recommended by Dr. Macbride of Dublin, of putting issues into the thighs in cases of Angina Pectoris, I thought it worth while to try whether relief could not be obtained from this remedy for these symptoms in cases of Anemia Africanorum. The first case in which I tried them was a young man who had had the complaint for a short time, and arising from causes I could not ascertain; pea issues were put into the middle of the inside of each thigh, and, as soon as the drain was properly established, great relief was experienced, and in five weeks the disease permanently disappeared. I have since employed them in many cases of this disease with very great advantage, having uniformly found these distressing symptoms to be much alleviated by them. A child two years of age, whom I scarcely expected to live sufficiently long for the establishment of an issue, derived the greatest benefit; the issues were kept up for about two months; he completely recovered, nor has he had any return, though the case occurred more than two years ago. I had several others who got well under this treatment alone, but of late I have combined them with the use of the rust of iron, and a shower-bath two or three times a week.

"Darwin expresses it as not very easy to explain the theory of the effect of issues in Angina Pectoris. I am equally at a loss to account for it in the cases before us, but as an empirical remedy, I have derived great assistance from them, and believe others may do the same. They have one inconvenience: Negroes do not like the trouble, and must be constantly watched, lest they allow the issues to dry up." (P. 70.)

Dr. Miller is of opinion that the resin and dried chips of guaiacum are almost inert, but that a decoction prepared from the wood when full of sap, is highly efficacious in venereal distempers.

Dr. Robert Bruce narrates a case of laceration of the abdomen with protrusion of the intestines, the patient having run against one of the cattle drawing a "trash-cart." The author says that "almost all the intestines and part of the omentum had escaped through the wound, and lay by her side, covered with cane-trash, sand, cinders, &c." (P. 72.) This happened on the 7th of January, 1834. The following is the termination of the case.

"11th, seven A.M. Much relieved; had several free evacuations per anum. I now directed my attention to the opening in the colon, and found adhesion had taken place between the gut and the parieties of the abdomen; brought the edges of the wound (including the gut) together with two stitches, adhesive plaster and bandage. The healing process went on very kindly; she recovered rapidly from this time, and in ten days was sent home. I did not see her afterwards, and I regret to say she died about six weeks after her removal (as I am informed) from fever." (P. 72.)

After a case of scrofula cured with iodine by Dr. M'Dermott, we come to the Editor's account of an epidemic variola, which appeared at Kingston in 1831. He mentions a case in which

"Such was the solution of the solids, that the scapular extremity of each clavicle had separated from its attachment, and had worked its way through the flesh, about three inches from each shoulder, where they appeared from an inch and a half to two inches, naked and dead bones, denuded even of the periosteum. Both trochanters were exposed, and the knee-joints were in the same state; indeed no limb could be raised without fear of separating its attachments. The flesh was of a livid colour, discharging a thin sanies, having the appearance of animal food in the last stage of putrescency; yet this object retained life, I believe for some days after I saw him." (P. 78.)

Mr. Maxwell has contributed a paper on acute traumatic tetanus, and trismus nascentium. The following were the post-mortem appearances in three cases of traumatic tetanus.

CASE I. A Negro aged twenty-two.

"The hard constricted state of the abdominal muscles disappeared when he became cold. The viscera of the thorax and abdomen were sound. The wound of the scalp was pale and flabby, neither the pericranium nor skull were injured. The substance of the brain was of a natural appearance, the right ventricle contained about a teaspoonful of serum; dura mater throughout its whole extent, but more especially towards the basis of the brain, exhibited a singularly beautiful and highly injected appearance, of a dark red colour, with here and there tortuous vessels unusually gorged with black blood. The cerebellum was minutely examined, but presented no deviation from the sound state; the nerves originating from the cerebrum, cerebellum, and medulla oblongata, were individually dissected, without any remarkable appearance being discovered. The cavity of the spine was opened from the back part, and the covering of the spinal marrow was found to be highly vascular, suffused, and turbid: the nervous mass, with the nerves, were raised and examined, but no morbid alteration was observed in this stage of the inspection—indeed the only unnatural appearances detected were in the involucra of the brain and spinal chord, which presented an intensely vascular looking anatomical preparation." (P. 82.)

Case ii. A Negro aged twenty-eight.

"The dura mater turgid with blood; between this membrane and the pia mater there was a general serous effusion which extended over the surface of the brain, nothing particular seen in the cortical or medullary substance, and the brain was of its natural firmness. The ventricles were filled with greenish serum, and the basis of the brain exhibited appearances analogous to those upon the upper surface; the crura cerebri enveloped in a highly vascular covering. The cerebellum was inundated with serum, and on separating the medulla oblongata from the medulla spinalis, the same fluid issued from the spinal canal in profusion. The spinal canal was opened and its contents examined, but with the exception of the unusual vascularity of the sheath, and disposition of serum, nothing else particular was observed.

"The abdominal cavity was inspected, and all the viscera found to be sound." (P. 84.)

Case iii. A stout healthy seaman.

"The body was opened six hours after death, by my assistant, Mr. Rapkey, and the following appearances noted. The skin had generally a purple appearance, like a stage in ecchymosis, but was not swollen. The contents of the abdomen were sound, with the exception of the liver, where a few tubercles were found; the thorax showed more disease; the pleura pulmonalis adhered to the pleura costalis; the lungs appeared turgid, and a few well defined tubercles were observed in their parenchyma. On removing the calvarium, the dura mater was particularly vascular, and the vessels felt like hard threads under the finger; about four ounces of black blood oozed from the longitudinal and lateral sinuses. On removing the dura mater, there was an appearance like a successful injection of blue and scarlet, where the pia mater dipped among the convolutions of the brain, the medullary part of which was studded with minute bloody points; the ventricles were distended with bloody serum; the brain was highly congested, and heavier and firmer than natural. The cerebellum, medulla oblongata and spinal marrow, exhibited appearances similar to those observed in the brain." (P. 85.)

The trismus nascentium is common among negro infants, and was formerly still more so. Our author considers it a kind of traumatic tetanus, and attributes it to improper treatment of the navel, together with the administration of stimulating medicines, and a vitiated atmosphere in the lying-in room. The following passages will show what very bad management and a tropical climate can effect in conjunction.

"A few years ago, when African customs predominated, and when it was morally impossible to reason or endeavour to convince them of an impropriety, I have often seen a tumbler full of equal parts of strong rum and castor oil, standing by the bedside, and when I have asked for what purpose it was to be used, they uniformly replied, that it was to feed the baby with till the ninth day. During this period, the air was carefully excluded from the room; a fire was kindled, and the midwife inculcated the necessity of not allowing the navel to be dressed till it dropped spontaneously. Under such treatment was it to be wondered at, if miliary eruptions, febrile symptoms, and strong convulsions should occur?" (P. 86.)

"A lady connected with the management of a plantation, had lost twenty-five infants in a few years from locked jaw, and as the women were delivered in one of the airy apartments of the dwelling-house, under her immediate superintendance, she was at a loss to know the cause of her misfortunes. Her treatment was to give the child a dose of oil shortly after birth, to dress the navel with bark, and each day to add a fresh portion to absorb the acrid matter without bathing till it dropped.

"I advised her to change her method of dressing the funis, to *bathe* it daily with milk and warm water, (or twice if it was large,) and apply a pledget of cerate or spermaceti ointment, and from adopting this simple plan of treatment she lately informed me that for several years she has never lost a child. Enough has already been said of the prevention of the disease, and never having seen a case cured, I shall now proceed to describe the appearances found on dissection.

"A woman on Fort Stewart Estate was delivered, in very easy labour, of a mulatto child. On the morning of the seventh day, the infant commenced crying, and shortly afterwards refused to take the breast, the fists now became clenched with partial spasms, which soon terminated in general tetanic convulsions and locked jaw, and the child expired next morning.

"*Dissection four hours after death.* The navel was unusually large, and did not drop till the commencement of the fits; the boy was well formed and born to the full time. On viewing the body externally, a blush of purple-red was observed suffused over several parts of the abdomen and back. The pericranium presented an injected appearance, and on slitting it open a coagulum of blood was found effused immediately over the junction of the parietal with the occipital bones. The dura mater was exceedingly vascular, pia mater turgid, and the corpora striata gorged with black blood; no lymph was found in the ventricles, but their lining was beautifully injected, and the right ventricle contained a small quantity of blood; tentorium and bases of the brain highly vascular. The cerebellum exhibited similar appearances to that of the brain.

"The spine was opened and carefully examined, involucre externally covered with blood, and internally with a slight effusion of blood and lymph; the appearances generally were highly vascular, but nothing further was observed throughout inspection than a preternatural injected appearance.

"A woman on Gray's Inn Estate brought a fine boy on Monday, after an easy labour; everything went on well till Friday, the fifth day, when about twelve, P.M., it appeared uneasy and gaped, shortly afterwards refused the breast, became restless, and was covered with profuse perspiration. A few hours later the jaw was found to be locked, and towards morning it was seized with convulsions.

"The whole of Saturday the fits recurred at intervals of two to three minutes, and were of a modified kind, but on Sunday they assumed an extremely violent form, and the head was now and then thrown back with great force, accompanied with dreadful spasms of the abdominal muscles, which became constricted and unyielding during the paroxysm; the breathing was hurried, and the arms were drawn upwards with clenched hands. The body was thrown into a general tremor, with pitiful moaning, and foaming at the mouth previous to the spasms. Died during the night.

"*Dissection a few hours after death.* Brain.—The dura mater was of a natural appearance, and presented no signs of inordinate vascular action. Pia-mater covered with turgid tortuous vessels, and had a minutely injected appearance. The sinus longitudinalis was gorged in the back part with coagulated blood. On making a horizontal incision through the middle of the cerebral substance, it was minutely studded with vessels which poured out drops of blood, the left ventricle was half filled with serum.

"The falciform process, tentorium, septum of the ventricles, and inferior dura mater, evinced signs of intense vascular action having existed, had a colour between vermilion and purple, and the depressions on the base of the brain were covered with layers of coagulable lymph. Cutting through the tuber, blood exuded from small points.

"Spine.—Dura mater morbidly suffused throughout its whole extent. The involucra of the lateral nerves of the same appearance.

"After inspecting the spinal chord very minutely in situ, it was carefully removed, and the nerves individually examined, and the only diseased changes observed during the whole dissection, were in the involucra of the brain and spinal chord, appearances which I have repeatedly noticed in many diseases not referable to spasmodic action.

"Abdomen.—The navel was inflamed and tumified, and an inflammatory blush extended a small way around it; the intestines were natural, but inflated; gall-bladder turgid, with dark green bile. Thorax healthy." (P. 87.)

There are some interesting papers in this Journal, on the best method of remedying that medical malady called Atrophy of the Purse, which appears to be effected in Jamaica by the external administration of unoxidized gold in very large doses. A couple of extracts will show that he who steals the purse of a Kingston doctor, does not steal trash.

"*Kingston; August* 1, 1834.

"We, the Undersigned, are of opinion that the time has arrived when the present mode of charging for our professional services should be abolished, as unworthy of an enlightened age, and a respectable profession.

"We are gratified also to know, that a similar opinion prevails in the Community.

"We do, therefore, now intend to relinquish the custom of charging for professional attendance by the Medicines that are prescribed; and we hereby make public this our intention, and also the scale of fees or charges by which we are for the future to be governed.

"*Scale of Fees.* The Medicines requisite in every case, will be supplied as heretofore, the charge being included in the fees.

"For the first visit daily, or when one *only* is required, the charge will be 20*s*., and for every succeeding visit on the same day, 10*s*. Not more than four visits to be charged on one day.

"For Domestics and Apprentices, where no agreement is made, two thirds of the above.

"For every night call, *viz.* betwixt the hours of nine, P.M., and six, A.M., 55*s*.

"For attendance at Pens (if above one mile from the Church), 13*s*. 4*d*. extra will be charged, and for every succeeding mile 13*s*. 4*d*.

"For attendance on board of Vessels, where no agreement is made, 26*s*. 8*d*. for each visit.

"Operations in Surgery, Midwifery, &c. &c., extra.

"When more than one, belonging to the same family, are ill in the same house, one half of the above fees will be charged for every additional patient.

"This arrangement will not interfere with any agreement now in existence.

"SPALDING & FERGUSON,
"CHAMBERLAIN & GARCIA,
"JAMES PAUL,
"GEO. K. PRINCE." (P. 106.)

Again, Mr. Downer, a country practitioner, in a letter to the Editor, offers his mite of information touching fees, to the Kingston Medical Committee, then and there investigating this most important subject, with the intention of establishing a tariff. He says,

"For attending an Express call, or paying a visit, my duty demanded, within or not exceeding a distance of five miles from my residence, my charge has been 1*l.* 12*s.* 6*d.*

"Ditto ditto ditto, beyond five miles, and not exceeding a distance of ten miles from my residence, 3*l.* 5*s.*

"For all night expresses, whether near to, or distant from my residence, my charge has never exceeded 5*l.* 6*s.* 8*d.*; and I may add, that I have attended many such even in dark nights, and over dangerous roads, to places distant from my house, from seven to ten miles.

"For the performance of all minor operations, such as venesection, extracting teeth, insertion of a seton, my charge has been extra, 1*l.* 12*s.* 6*d.*

"For arteriotomy, cupping, tapping, &c. &c., 3*l.* 5*s.*

"For the reduction of dislocations or fractures, amputations, dissections, common cases of midwifery, &c. &c., 5*l.* 6*s.* 8*d.*

"For prescribing for a patient attending at my house for advice, 16*s.* 3*d.*

"For all operations performed under the same circumstances, one half of the respective already stated charges.

"For remaining at a patient's house all day in close attendance, at the request of the patient, my charge has been, 2*l.* 13*s.* 4*d.*

"For ditto ditto all night, ditto ditto, 5*l.* 6*s.* 8*d.*

"Medicines are not usually furnished by practitioners in the country, but whenever I have been requested to do so from my own little dispensary, as a matter of accommodation to my patients, my charge for them has not exceeded such as would, under similar circumstances, have been made by the druggists of Kingston." (P. 111.)

Adam Smith remarks, that in spite of all that has been said about the roaming disposition of man, he is in truth the most difficult to transport of any kind of luggage, and a case in point may be built upon these colossal fees; for it would seem that so many prefer half-a-crown in Cornwall or Cumberland to a moidore in Kingston, that fees can remain on a very different level in these several spots.

The Jamaica Physical Journal is creditable to Mr. Paul, and we trust that it is prospering.

